# Author Correction: In vivo marker of brainstem myelin is associated to quantitative sleep parameters in healthy young men

**DOI:** 10.1038/s41598-024-56474-8

**Published:** 2024-03-13

**Authors:** Puneet Talwar, Michele Deantoni, Maxime Van Egroo, Vincenzo Muto, Daphne Chylinski, Ekaterina Koshmanova, Mathieu Jaspar, Christelle Meyer, Christian Degueldre, Christian Berthomier, André Luxen, Eric Salmon, Fabienne Collette, D.-J. Dijk, Christina Schmidt, Christophe Phillips, Pierre Maquet, Siya Sherif, Gilles Vandewalle

**Affiliations:** 1 GIGA-Institute, CRC-In Vivo Imaging Unit, Bâtiment B30, Université de Liège, 4000 Liège, Belgium; 2 Walloon Excellence in Life Sciences and Biotechnology (WELBIO), Wallonia, Belgium; 3 Psychology and Cognitive Neuroscience Research Unit, University of Liège, Liège, Belgium; 4 Physip, Paris, France; 5 Sleep Research Centre, University of Surrey, Guildford, UK; 6 UK Dementia Research Institute, University of Surrey, Guildford, UK; 7 In Silico Medicine Unit, GIGA-Institute, University of Liège, Liège, Belgium; 8 Department of Neurology, CHU of Liège, Liège, Belgium; 9 Present Address: Faculty of Health, Medicine and Life Sciences, School for Mental Health and Neuroscience, Alzheimer Centre Limburg, Maastricht University, Maastricht, The Netherlands

Correction to: *Scientific Reports* 10.1038/s41598-023-47753-x, published online 27 November 2023

The Acknowledgements section in the original version of this Article was incomplete. It now reads:

“PT and LL are supported by the EU Joint Programme Neurodegenerative Disease Research (JPND) IRONSLEEP and SCAIFIELD projects, respectively – FNRS references: PINT-MULTI R.8011.21 & 8006.20). MD, MVE, EK, FC, CS, CP and GV are/were supported by the Fonds de la Recherche Scientifique - FNRS-Belgium. The study was supported by the Wallonia-Brussels Federation (Actions de Recherche Concertées - ARC—09/14-03), WELBIO/Walloon Excellence in Life Sciences and Biotechnology Grant (WELBIOCR-2010-06E), FNRS-Belgium (FRS-FNRS, F.4513.17 and T.0242.19 and 3.4516.11), Fondation Recherche Alzheimer (SAO-FRA 2019/0025), University of Liege (ULiege), Fondation Simone et Pierre Clerdent, European Regional Development Fund (Radiomed project), Fonds Leon Fredericq, EU JPND program (IRONSLEEP project - PINT-MULTI R.8011.21), ULiège-Valeo Innovation Chair "Health and Well-Being in Transport" and Siemens. D.J.D. is supported by the UK Dementia Research Institute (DRI). SS was supported by the ULiège-Valeo Innovation Chair and Siemens Healthineers. We acknowledge Christian Lambert for providing the scripts to perform the brainstem segmentation. The authors also thank Christine Bastin, Annick Claes, Catherine Hagelstein, Gregory Hammad, Brigitte Herbillon, Patrick Hawotte, Sophie Laloux, Erik Lambot and Benjamin Lauricella for their help over the different steps of the study. This work was conducted at the GIGA-CRC-Human Imaging platform of ULiège, Belgium.”

The original Article has been corrected.

